# The Conversion of a Practice-Based Lifestyle Enhancement Program into a Formalized, Testable Program: From Texercise *Classic* to Texercise *Select*

**DOI:** 10.3389/fpubh.2014.00291

**Published:** 2015-04-27

**Authors:** Marcia G. Ory, Matthew Lee Smith, Doris Howell, Alyson Zollinger, Cindy Quinn, Suzanne M. Swierc, Alan B. Stevens

**Affiliations:** ^1^Department of Health Promotion and Community Health Sciences, Texas A&M Health Science Center School of Public Health, College Station, TX, USA; ^2^Department of Health Promotion and Behavior, College of Public Health, The University of Georgia, Athens, GA, USA; ^3^Big Brothers Big Sisters of South Texas, San Antonio, TX, USA; ^4^Baylor Scott & White Health, Temple, TX, USA

**Keywords:** translational research, program implementation, program evaluation, older adults, physical activity

## Abstract

Little is known about the structure, content, and benefits of practice-based or grass roots health programs that have been widely delivered by a variety of community organizations and stakeholders. This perspective will document the natural history of Texercise *Classic*, a state-endorsed but previously untested lifestyle health promotion program. It will: (1) discuss Texercise *Classic’s* participant reach and adoption over time; (2) describe the rationale and processes employed to formalize Texercise *Classic* into a more structured program known as Texercise *Select*; (3) outline the essential elements and activities included in Texercise *Select* and contrast them with those included in Texercise *Classic*; and (4) highlight key components for uniform facilitator training. The discussion will reflect upon the evolution of Texercise, compare and contrast the benefits and challenges of each program, and review the “next steps” for Texercise *Select*. In contrasting Texercise *Classic* and *Select*, it is important to understand the benefits and challenges of both programs. Preliminary results indicate that Texercise *Select* is effective, yet its ability to sustain the same reach as Texercise *Classic* remains unknown and an area for future study.

## Introduction

With greater recognition of the value of health promotion for adults across the life-course (1), a multitude of programs now exist to improve health and wellness among older adults (2, 3). These programs recognize that older adults are able and willing to engage in health promotion programs and can derive substantial health benefit from those programs (4). There have been two programmatic streams to meet the needs of the rapidly growing population of older adults: (1) practice-based or grass roots programs promoted by community-based organizations; and (2) research-tested programs developed and tested in academic research centers.

Included in the first programmatic stream, health promotion/disease prevention programs have traditionally been delivered by non-academic community-based practitioners with the generic goals of maintaining or improving health (5–8). Because these programs are delivered in real-world settings, they have a greater potential for large population reach and long-term sustainability; however, they are typically unstructured and hence not easily replicable or testable. Further, even when based on “best practices,” such programs may have minimal attention to behavioral change theories and little to no formal evaluation to document intervention effectiveness or characterize essential program elements (9–12).

A second programmatic stream involves the recent nationwide movement toward the widespread dissemination of evidence-based programs (EBPs) for older adults that were developed and tested by academics in controlled settings. This movement reflects the assumption that EBPs are preferable because they are “assumedly” more efficient and cost-effective than programs that are not theoretically based and rigorously evaluated (13). With the initiation of the Administration on Aging (AoA) Evidence-based Disease Prevention Initiative (14, 15), substantial knowledge has been gained about the nature and effectiveness of EBPs for older adults, especially those related to fall prevention, chronic disease self-management, and specific lifestyle behaviors such as physical activity or healthy nutrition (16–18).

Less is known, however, about the structure, content, and benefits of grass roots health programs that have been delivered by a variety of community organizations across vast geographic distances (i.e., regional or state-wide health promotion campaigns or community walking programs). Further, little attention has been given to understand how these programs might contribute to practice-based evidence. Given the recent emphasis on administrative policies within the Administration on Community Living (ACL), which give funding preference to EBPs (19), it is especially important to understand how these long-standing, community-based health promotion programs have functioned in the past and how they might be adapted to permit formal evaluation and be eligible for governmental funding streams.

This perspective article presents a case study of Texercise, a community-based health promotion movement established in 1999 to help Texans ages 45 and older live healthier lives. Based on a historical review of existing Texercise materials, supplemented by information provided by key Texercise staff, this article will examine the processes employed to structure the original Texercise program (referred to as Texercise *Classic*) so that it could be formally evaluated. Specifically, this article will: (1) document the natural history of Texercise *Classic*, including its programmatic reach and adoption across Texas over the past decade and a half; (2) describe the rationale and processes employed to formalize Texercise *Classic* into a more structured program known as Texercise *Select*; (3) outline the essential elements and activities included in Texercise *Select* and contrast them with those included in Texercise *Classic*; and (4) highlight key components for uniform facilitator training. The discussion will reflect upon the evolution of Texercise, the comparisons across the two program types, and the “Next Steps” for Texercise *Select*.

## Natural History

Texercise *Classic* emerged from a vision by the state public health and aging services leadership to help the growing number of Texans age well. We highlight some salient events in the development and evolution of Texercise. Starting as a state-wide public health campaign in the late 1990s, this grass roots program was officially launched in 2002 as part of the Governor’s Challenge Walk for Wellness. Under the Governor’s Office, Texercise was envisioned as a state-wide health promotion program to encourage individuals and communities to adopt healthy lifestyle habits such as physical activity and good nutrition. In 2005, strong endorsement was received from the Governor’s Office through Executive Order (20), which stated that “The Department of Aging and Disability Services, Department of State Health Services, Governor’s Advisory Council on Physical Fitness, and other appropriate state and community organizations shall continue to promote and expand the internationally recognized Texercise program as a means to ensure healthy lifestyles in older Texans.” In 2006, under the auspices of the Texas Department of Aging and Disability Services (DADS), Texercise became more formalized with the creation of a 12-week face-to-face fitness program, with the tag line “*Fit for the Health of It!*”, and the identification of community-based volunteers or “program champions” to promote Texercise. In 2009, with input from experts such as Dr. Kenneth Cooper from the internationally known Cooper Clinic (21), the 12-week program and materials were updated to include attention to both physical activity and nutrition. The basis for the nutritional content was the existing information sheets developed by nutrition experts at DADS. We also utilized some standard nutrition items that other EBPs employ as well, such as the USDA my plate, and had all materials reviewed by a nutrition expert at the Texas A&M Health Science Center School of Public Health.

Thanks to the collaboration between DADS and its partners, what has become known as Texercise *Classic* is available free-of-charge and includes resources and incentives such as pedometers, resistance bands, pledge sheets, and 12-week daily fitness and nutrition logs distributed to participants during the program. Using a lay-leader facilitator model, Texercise *Classic* has been delivered through a variety of settings including worksites, senior centers, faith-based organizations, and long-term care facilities.

Texercise *Classic* has reached more than 15,000 Texans starting with 794 participants in 2006 and growing to 3,400 participants in 2012. Further, since 2003, over 160,000 Texercise handbooks (available at www.texercise.com) about how to initiate an exercise program have been distributed to individuals wanting to exercise on their own (22). Despite its widespread reach across Texas and national recognition (e.g., the International Council on Active Aging Industry Innovator Award; President’s Council on Fitness, Sports, and Nutrition Community Leadership Award; and the Texas Cardiovascular Health Promotions Award), Texercise *Classic* had never been formally evaluated.

In 2012, a contract was awarded to the Texas A&M Health Science Center in collaboration with Baylor Scott & White Health to review and evaluate the program. The primary aims of this contract were to formalize the processes and procedures (including materials and facilitator training) and collect more detailed information from participants with the hopes of establishing a rigorous, scientific evidence base for this program. This evaluation has generated a new phase of activity.

## Formalization Processes and Procedures

### Process overview

We identified in the beginning stages of the evaluation project that the loosely structured nature of the existing Texercise *Classic* program would make program evaluation difficult. Initially, Texercise *Classic* was designed as a participant-driven grass roots program in which participants, in collaboration with group leaders, decide upon the nature and amount of group exercises. While this strategy offered substantial choice, the lack of uniformity between workshops offered made it difficult to examine effectiveness and generalize to all Texercise programs. The Texas A&M research team and DADS staff jointly decided to utilize existing program materials and activities to create a more formally designed program. As indicated in Table 1, the resulting “structured” program is known as Texercise *Select*. Texercise *Select* is implemented in 12 weeks, which includes 2 weeks for participant recruitment and 10 weeks of 1.5-hour sessions conducted twice a week. Utilizing evidence-based skills and tools, each session incorporates interactive educational discussions, interactive activities related to physical activity and/or nutrition topics, and 30–45 minutes of actual exercise.

**Table 1:** **Texercise *Select* topics, objectives, and resources by week**.

Week	Sessions and topics	Objectives
1 & 2	None: participant recruitment	
3	Session 1: Ready, Set, Get Active: Launching an Active Lifestyle	Describe the 11 principles of physical activity success
		Identify their personal exercise levels
		Understand the importance of a warm-up and cool-down
		Set realistic goals related to physical activity
	Session 2: Ready, Set, Eat Healthy! Healthy Eating for a Healthy Lifestyle	Understand the benefits of healthy eating as well as the nutritional components of a healthy diet
		Make an achievable and realistic nutrition goal
		Describe the purpose of a nutrition log
4	Session 3: Ready, Set, Get Moving! Getting & Staying Physically Active	Recognize the essential components of being and staying physically active
		Practice endurance, strength, balance, and stretching exercises safely and correctly
		Create an action plan
	Session 4: Ready, Set, Eat Healthy! Eating a Balanced and Healthy Diet	Recognize the essential components of a balanced and healthy diet
		Practice new exercises safely and correctly
		Select and incorporate five sources of fruits and vegetables into their diets
5	Session 5: Ready, Set, Hydrate! Hydration for Health	Explain the basis and requirements of proper hydration
		Practice previous exercises safely and correctly
		Identify barriers and apply problem-solve skills when action planning
	Session 6: Ready, Set, Eat Proper Portions! Establishing a Sense of Portion Control	Understand healthy portion sizes for most types of foods
		Practice new exercises safely and correctly
		Identify ways to eat sensible portions
6	Session 7: Ready, Set, Go Endurance! A Focus on Endurance	Identify ways to safely increase endurance
		Practice exercises safely and correctly
		Evaluate previous action plan and apply strategies to overcome challenges with personal action plans
	Session 8: Ready, Set, Decode Food Labels! Understanding Food Labels	Explain the fundamental components of a food label
		Practice new exercises safely and correctly
		Evaluate dietary logs and action plans and apply strategies to overcome challenges
7	Session 9: Ready, Set, Prevent Injury! Injury Prevention for Better Health & Safety	Identify and apply injury prevention methods before, during, and after physical activity
		Practice safe and correct exercises
		Evaluate previous action plans and apply strategies to overcome challenges
	Session 10: Ready, Set, Cook Healthy! Cooking Healthy for Improved Nutrition	Identify and apply healthy cooking modifications to maximize nutritional intake
		Practice new exercises safely and correctly
		Recognize challenges and apply strategies to improve dietary behaviors
8	Session 11: Ready, Set, Get Strength Training	Understand and apply the fundamentals of strength training introduced in class
		Practice exercises safely and correctly
		Evaluate previous action plans and challenges
		Identify and apply strategies to overcome challenges with personal action plans
	Session 12: Ready, Set, Eat Out Healthy: Eat Healthy When Dining Out	Identify and apply strategies to make healthy choices when eating outside the home
		Practice exercises safely and correctly
		Create a healthy meal or menu
9	Session 13: Ready, Set, Don’t Stress! Stress Management & Mental Health	Recognize and discuss healthy behaviors that reduce stress
		Practice exercises safely and correctly
		Evaluate previous actions plans
	Session 14: Ready, Set, Prevent Chronic Illness! Healthy Preventive Behaviors	Identify and select strategies to overcome challenges with action plans/goals
		Recognize and discuss healthy behaviors
		Practice exercises safely and correctly
		Identify ways to prevent and better manage chronic illnesses
10	Session 15: Ready, Set, Keep Fitness Fun! Keeping Fitness Fun	Practice exercises safely and correctly
		Identify ways to make long-term fitness enjoyable
		Identify and select strategies to overcome challenges with action plans/goals
	Session 16: Ready, Set, Eat Healthy! Eating Healthy During the Holidays	Identify healthy eating alternatives during the holidays
		Practice exercises (safely and correctly)
		Recognize unhealthy eating habits
11	Session 17: Ready, Set, Stay Committed! Staying Committed to Fitness & Review	Practice exercises safely and effectively
		Apply the two-step approach to creating an action plan
		Identify and apply strategies to overcome challenges
		Identify and apply safe ways to stay physically active (review)
	Session 18: Ready, Set, Stay Nutritious! Keeping Nutrition a Lifestyle & Review	Identify and apply ways to stay committed to nutritional goals and healthy eating
		Practice new exercises safely and correctly
		Identify and apply skills maintaining a healthy lifestyle
12	Session 19: Ready, Set, GO! Moving Forward Successfully	Identify and apply ways to stay physically active and eat nutritiously
		Practice exercises safely and correctly
		Identify and apply strategies to overcome barriers of physical activity and healthy eating
	Session 20: Ready, Set, CELEBRATE!	Identify and apply ways to stay committed to physical activity nutritional goals and healthy eating
		Demonstrate exercises safely and correctly

### Essential intervention elements

Pulling from foundational concepts in evidence-based health and wellness programs (23–26), the research team developed the “structured” Texercise *Select* program, manual, and training that operationalized essential intervention elements and processes. To accomplish this task, the research team reviewed the literature as well as comparable EBPs. This review enabled the team to identify key exercise and behavior change elements that would work best in the Texercise context (e.g., ideal session length, ideal class duration, and types of effective exercises). Drawing on social cognitive learning principles (27), the entire program was designed heavily around the concept of self-efficacy with a goal of having participants take a more active role in their health through health choices and behaviors. The underlying programmatic intent was to increase self-efficacy and behavioral skills so that participants would continue to engage in healthy aging activities after the program ended.

Texercise *Select* sessions were organized around a “Ready, Set, Go, Stay” rubric developed by our our Texas A&M program designers to help participants know what they needed to do to initiate healthier behaviors, engage in those behaviors, and to make them part of their everyday routines. Typically, each session has a physical activity and nutrition educational component, with the exception of the weekly session that focused more generally on managing emotional issues and general lifestyle behaviors. Using handout materials that had already been developed by experts for Texercise *Classic* (22), the course activities were intended to help participants apply strategies for enhancing healthy lifestyle behaviors. For example, group brainstroming was utilized to help participants identify solutions to common barriers. Class instructors also taught participants the essence of action planning – identifying, setting, and implementing realistic goals.

Although the program was structured (e.g., class length, discussion topics, and types of exercise specified), it was designed to be highly participatory and interactive with participants learning to actively engage in behavior change principles such as goal setting, problem solving, tracking behaviors, and providing support to fellow class participants. In addition to a peer-to-peer learning approach, Texercise *Select* was built around a lay-leader model, which has proven highly successful in the delivery of other EBPs for older adults (17, 28–30). This is consistent with the new exercise guidelines for older adults that stress the importance of risk management in the delivery of physical activity programs (25, 26, 31). Formal training sessions (i.e., a 6-hour group training) were hosted by the research team to provide lay-leaders (known in Texercise *Select* as facilitators) with information about how to safely introduce exercises. These training sessions were supplemented with course material that included screening questions and safety tips for participants (32).

Given the high probability of behavioral relapse in achieving one’s desired lifestyle behavioral goals (24), the curriculum was designed for 10 weeks to increase the likelihood that behaviors would be adopted and become habit through ongoing reinforcement. It included attention to explicit strategies for helping participants stay committed. This involved hands-on practice of different behavioral skills (e.g., goal setting) combined with discussion about ways to overcome barriers and meet physical activity and healthy eating goals. Additionally, participants were encouraged to incorporate more walking into their daily routines and use the Texercise workout DVD at least 1 day each week outside of class (reinforced by program facilitators at the conclusion of each session).

### Comparison of Texercise *Classic* and *Select*

Table 2 compares the elements of Texercise *Classic* with those of Texercise *Select*. When compared to Texercise *Classic*, Texercise *Select* has some similarities and many substantial differences. We draw upon Schulz and colleague’s (33) taxonomy of interventions to describe some of the most prominent similarities and differences. When Texercise *Classic* was first designed, less was known about best practices for exercise training for older adults, and the program concepts were more implicitly related to best practices (rather than explicitly related to best practices). In contrast, Texercise *Select* was designed by individuals with formal training in exercise science and behavioral science as related to older adults. As such, this version of the program has benefited from an emerging science and practice base in both of these disciplines (26). Additionally, when creating Texercise *Select*, the developers drew upon the RE-AIM and other public health frameworks (34–36), for understanding the importance of key implementation and dissemination elements such as maximizing population reach, adoption, implementation, and sustainability.

**Table 2:** **Comparison of features in Texercise *Classic* and *Select***.

	Texercise *Classic*	Texercise *Select*
**THEORETICAL UNDERPINNINGS**		
Built around best practices for exercise training	Not explicit, but implicit through endorsement of Dr. Kenneth Cooper	Yes, using ACSM for older adult guidelines
Built upon best practices for behavioral change	Actual theoretical basis not clearly stated	Social cognitive theory (self-efficacy and other behavior change principles)
		RE-AIM framework
		Diffusion of innovation
**PROGRAM STRUCTURE, APPROACH, AND POPULATION TARGET**		
Total program duration	12 weeks of chosen exercise/activity	12 weeks: 2 weeks of recruitment plus 10 weeks, 2×/week of actual sessions equals 20 sessions total
Number of weeks of active intervention	12 weeks of chosen exercise	10 weeks, 2×/week of actual classes equals 20 sessions total
Amount of time per class	Variable	Structured – 90 minutes
Sensitivity to participant characteristics	Can be lay lead, leaders can be representative of the participants	Can be lay led, leaders can be representative of the participants
	Participants do not need to be cognitively able to understand possible educational components	Participants must be cognitively able to understand the educational component including action planning
	Spanish materials are available	Not yet translated to Spanish
**PROGRAM DESIGN**		
Intervention manual	General guidance with class instructors are only given the Texercise pink packet that includes:	Structured manual for program facilitators with detailed session outlines
	Promotional DVD	
	Texercise handbook	
	Pledge sheets	
	Incentives (pedometers, t-shirts, etc.) and have access to the online resources	
Adaptability	All aspects can be adapted, except involving some sort of PA	Exercises can be adapted to participant level of PA
	Anyone can make an adaptation to the program including sites and class leaders	Field coordinators/class facilitators cannot make adaptation to essential features of the program
	Adaptations can be made at any time	
**PROGRAM CONTENT**		
Attention to physical activity and nutrition	Possibility with the fact sheets	Built into the program
	Not necessary or monitored to see if the info provided to participants is factual	First session of the week deals with a physical activity topic
		Second session of the week deals with a nutrition topic
Use of information sheets	Optional	Integrated into class curriculum
Opportunity for engaging in in-class exercises	Yes	Yes
Recommended exercises	Variable	Drawn from prescribed list with goal of 30–45 minutes of exercise per session that must include flexibility, strength, balance, and endurance
Opportunity for interactive class discussions on goal setting and problem solving	Due to the variability of the classes this is unknown	Utilizes action planning and brainstorming
	Goal setting and problem solving are not specifically addressed in the classic class	Physical activity and dietary logs are kept through the first half of the sessions
		Uses incentives for behavior change
		Tracking and monitoring behavior (logs)
		Teaches problems solving
		Provides skill building (i.e., learning exercises)
		Provides social support
**TRAINING AND EVALUATION**		
Training of instructors	Variable	Structured – 1 day 6 hour training
Pre-post assessment	None	Part of curriculum
Fidelity monitoring	None	On-site class fidelity checklist
		Post class survey for participants

Although both programs are viewed as 12-week programs (with 10 weeks of active programing), the structure varies across the two programs, which makes the actual program duration and class time likely to vary as well. Texercise *Classic* and *Select* both provide time for participants to engage in group exercises and demonstrate some sensitivity to individual participant’s needs, preferences, and level of physical functioning. One primary difference relates to program flexibility in that Texercise *Classic* operates under general guidance in contrast to Texercise *Select* that has a detailed implementation manual, which limits any adaptation to essential program characteristics or general program flow. Given the flexibility in structure and lack of detailed facilitator manual, it is assumed that Texercise *Classic* attends less consistently to both physical activity and nutrition aspects of healthy living. Texercise *Classic* was also assumed less likely to utilize the information sheets, demonstrate specific exercises, and promote interactive class discussions about goal setting and problem solving. However, the extent to which this is true is unknown because Texercise *Classic* has never been formally evaluated. Finally, the two programs differ in instructor training, evaluation, and fidelity monitoring with only Texercise *Select* including a pre- and post-assessments and a fidelity checklist as part of the program. While Texercise *Select* was evaluated as part of a research study, it should be noted that the capacity for on-site fidelity check monitoring in grand scale dissemination efforts may be limited.

### Texercise facilitator training

When developing Texercise *Select*, the research team decided to utilize the term “facilitator” for program “lay” or “peer” leaders who are typically community volunteers versus health professionals. This decision was intended to emphasize that facilitators are not experts; rather, their role was to “facilitate” participants’ ability to influence their health and functioning by presenting them with the concepts and exercises included within the program. The facilitator training was seen as essential for maintaining treatment fidelity (37, 38). Total facilitator training time consisted of one 6-hour day, with a structured training manual to which facilitators could refer after training. As specified in the training manual (39), the facilitator training was divided into five main topic blocks, each lasting between 30 minutes and 1.5 hours. Topics were delivered by the Texas A&M trainers through an interactive lecture style, including activities that allowed facilitators to apply presented information and elicit group feedback.

The training included a brief program overview of Texercise *Select* and an introduction to the format of each session in the curriculum. The training provided an opportunity for facilitators to observe and practice selected exercises, as well as to observe and demonstrate their ability to engage in group facilitation roles. During the demonstration session, trainers played the roles of the facilitators during a session and facilitators played the role of participants.

To demonstrate competency to lead a Texercise class, facilitators were assigned a Texercise session and tasked with four activities: (1) identifying the session topic; (2) identifying session materials needed; (3) identifying session activities; and (4) choosing one exercise from each exercise category (i.e., one warm-up, one upper body, and one lower body strength activity) and demonstrating it. Facilitators were then critiqued by trainers and any issues discussed and clarified.

Twenty-nine facilitators were trained during this pilot study. The curriculum was originally developed with two Texas A&M trained facilitators per class in mind. Once the research team began working with organizations to identify implementation sites and facilitators, it became apparent the class would most likely be led by one trained facilitator with assistance from another person who had not gone through the formal A&M training session. Given the pilot nature of this demonstration study, the Texas A&M class trainers were available by telephone and email to provide additional assistance to newly trained facilitators.

### Summary of evidence-based steps

We employed several steps in transforming *Texercise Select* into a testable and replicable EBP. These involved: (1) inventorying the current literature to identify foundational concepts in evidence-based health and wellness programs, with special emphasis on strategies for promoting participant’s self-efficacy for engaging in physical activity and peer-to-peer learning; (2) evaluating the match between existing programmatic elements and anticipated delivery capacity and structure to ensure program adoptability and maximal population coverage; (3) organizing the sessions around a “ready, set, go, stay” framework for ease of implementation; (4) developing a standardized manual and training protocol; (5) incorporating fidelity checks and quality assurance into the implementation and evaluation processes; and (6) identifying a practical measurement battery to assess pre–post intervention outcomes.

## Discussion

As indicated in our brief historical review, the evolution of Texercise mirrors many of the critical steps taken during the development and evaluation of an evidence-based health promotion program. This review also demonstrates the interaction between state-wide policy priorities, community practice, and research. Unlike research-based programs that often struggle for scalability, its state governmental sponsorship made Texercise *Classic* widely available and disseminated through existing community partners and delivery systems even before it was formally evaluated. Further, participation in Texercise *Classic* has grown state-wide for over 15 years, confirming the importance of high level endorsements and community buy-in for achieving long-term program sustainability (40–42). Its relatively low cost and use of volunteer networks have also been probable factors in its successful dissemination (43).

Although there have not been systematic studies, it is likely that Texercise *Classic* gained steam, in part, because it was endorsed by the Governor, codified in executive orders, supported for implementation as part of state services, and stimulated through active encouragement of public–private partnerships. Texercise *Classic* grew from a public health campaign with community-friendly handouts to a face-to-face group program based on best practices and expert opinion. As evolved by science, Texercise *Classic* progressed from an exercise program focused on physical activity to a behaviorally based program including attention to both physical activity and nutrition. Additionally, over time, with the movement toward evidence-based programing with replicable and demonstrated effects, the program was redesigned as Texercise *Select* to include explicit attention to best practices about exercise training and behavioral change found in other successful lifestyle programs that meet the highest tier criteria Evidence-based Disease Prevention and Health Promotion Program (19).

The development of new programs or the formalization of existing programs can expand the evidence base as it pertains to older adult health and wellness. While community-generated programs such as Texercise *Classic* may have already demonstrated success based on their reach and adoption, new policies from federal funders in the U.S. aging services sector are restricting reimbursement to reproducible health promotion programs with proven benefits (16, 19). As such, this case study illustrates the processes and procedures involved in the formalization of this community-generated program to advance its sophistication, replicability, and likelihood of evoking health benefits among its participants. To mirror requirements for EBP status, Texercise *Select* is now characterized by a set of essential features including formal manual and training infrastructure for widespread delivery (44, 45). Thus, the process described in this review represents the first steps toward formalizing Texercise *Select*, which is undergoing systematic evaluation to examine program effectiveness. Looking forward, the RE-AIM framework (46) will be used as a guide for examining strategies for demonstrating Texercise *Select* program effectiveness and public health impact, especially around program implementation, scalability, and sustainability issues.

In contrasting Texercise *Classic* and *Select*, it is important to understand the benefits and challenges of both programs. Texercise *Classic* has demonstrated its widespread appeal and sustainability by continual delivery for more than ten years by volunteer facilitators who do so without external financial support. As indicated in interviews with stakeholders (47), it is a program that has name recognition, is easy to implement, and is well-liked by program facilitators and participants alike. However, its greatest strength – flexibility in the content and type of delivery – is also its greatest potential weakness as an EBP. Such flexibility makes it difficult to replicate consistently, know exactly what program components are being implemented, and measure the extent to which participants are benefiting (or in what ways). Specific outcomes are unknown but are likely to be quite variable and affected by individual delivery settings, facilitators, and participant populations.

Conversely, Texercise *Select* provides structured training for facilitators and a scripted curriculum that, if followed, should result in positive health outcomes similar to those of other evidence-based lifestyle programs. Yet, some existing partners who primarily offer exercise programs might not like (or be able to implement) the reconfigured program with fidelity. For example, some park and recreation programs might easily adapt the exercise training part but not be as comfortable with facilitating the behavioral lifestyle educational aspects.

However, in terms of program impact, Texercise *Select* is likely to be more effective than Texercise *Classic* in changing lifestyle behaviors because of its standardized incorporation of evidence-based behavioral change principles. Yet, it is also unknown whether Texercise *Select* will be as appealing to community organizations and able to sustain the same reach and adoption as Texercise *Classic*. This issue illustrates a potential trade-off often seen when attempting to simultaneously achieve public health reach and effectiveness (48), and remains an area for future study.

The research team was able to redesign Texercise and conduct standardized training within two months. This accelerated timeframe was possible because of the research team’s familiarity with EBPs and leader training as well as the insights provided by the original state-based developers of Texercise. Working together, opportunities, challenges, and potential solutions were identified. Texercise has a brand that is already established throughout the state with an existing network of partners. This brand was capitalized upon during the transition from Texercise *Classic* to Texercise *Select*. While a new name was considered for Texercise *Select*, the Texercise name was kept to ensure recognition and consistency. Both programs will continue to be promoted and supported in Texas because DADS sees value in allowing their partners to choose the appropriate program to offer based on their settings and participants.

## Next Steps

The purpose of this article was to illustrate the evolution of a grass roots program to become a theoretically derived and research-tested program. The further expansion of Texercise *Select* is dependent, in part, upon demonstrating positive outcomes comparable to those found in similar EBPs for seniors. An initial pilot test of the feasibility of implementation and outcomes was conducted in 2013. Preliminary results are promising (49, 50), with significant pre-post improvements (*P* < 0.05) seen in positive health behaviors (i.e., increased aerobic activity, weekly fruit/vegetable consumption, and daily water consumption) with large effect sizes for physical activity and smaller ones for nutrition behaviors. Additionally, enhanced dissemination of Texercise *Select* requires infrastructure resources such as the widespread availability of standardized training. Toward this end, DADS is updating the training and implementation manual so it will be web-based and easily accessible by community partners and potential facilitators. Although initial outcome results are promising, further study is needed to understand factors associated with the ability of Texercise *Select* to be widely disseminated and sustained over time. Once the results of initial pilot testing from 2013 are fully published, we recommend a state-wide campaign with DADS’s current Texercise partners to help spread the word about the benefits of implementing evidence-based programing for seniors and how Texercise *Select* might be broadly disseminated through existing community channels.

## Conflict of Interest Statement

The authors declare that the research was conducted in the absence of any commercial or financial relationships that could be construed as a potential conflict of interest.

This paper is included in the Research Topic, “Evidence-Based Programming for Older Adults.” This Research Topic received partial funding from multiple government and private organizations/agencies; however, the views, findings, and conclusions in these articles are those of the authors and do not necessarily represent the official position of these organizations/agencies. All papers published in the Research Topic received peer review from members of the Frontiers in Public Health (Public Health Education and Promotion section) panel of Review Editors. Because this Research Topic represents work closely associated with a nationwide evidence-based movement in the US, many of the authors and/or Review Editors may have worked together previously in some fashion. Review Editors were purposively selected based on their expertise with evaluation and/or evidence-based programming for older adults. Review Editors were independent of named authors on any given article published in this volume.
